# Natural phenolic compounds potentiate hypoglycemia via inhibition of Dipeptidyl peptidase IV

**DOI:** 10.1038/s41598-019-52088-7

**Published:** 2019-10-30

**Authors:** Po-Kai Huang, Shian-Ren Lin, Chia-Hsiang Chang, May-Jwan Tsai, Der-Nan Lee, Ching-Feng Weng

**Affiliations:** 1 Department of Life Science and Institute of Biotechnology, National Dong Hwa University, Hualien, 97401 Taiwan; 2 Neural regeneration Laboratory, Neurological Institute, Taipei Veterans General Hospital, Taipei, 11217 Taiwan; 3 Department of Biotechnology and Animal Science, National Ilan University, Ilan, 26047 Taiwan; 4 Faculty of Applied Sciences, Ton Duc Thang University, Ho Chi Minh City, Vietnam

**Keywords:** Virtual drug screening, Type 2 diabetes

## Abstract

Dipeptidyl peptidase IV (DPP IV) is a surface glycoprotein that can degrade glucagon like pepetide-1 (GLP-1) by decreasing blood sugar. Herbal medicines for diabetic therapy are widely used with acceptable efficacy but unsatisfied in advances. DPP IV was chosen as a template to employ molecular docking via Discovery Studio to search for natural phenolic compounds whether they have the inhibitory function of DPP IV. Then, docking candidates were validated and further performed signal pathway via Caco-2, C2C12, and AR42J cells. Lastly, a diet-induced diabetes in mice were applied to examine the efficacy and toxicity of hit natural phenolic products in long-term use (*in vivo*). After screening, curcumin, syringic acid, and resveratrol were found in high affinity with DPP IV enzymes. In enzymatic tests, curcumin and resveratrol showed potential inhibition of DPP IV. *In vitro* assays, curcumin inhibited of DPP IV activity in Caco-2 cells and ERK phosphorylation in C2C12 cells. Additionally, curcumin attenuated blood sugar in S961-treated C57BL/6 mice and in diet-induced diabetic ICR mice and long-term regulate HbA1c in diabetic mice. Curcumin targeted to DPP IV for reducing blood glucose, it possesses potential and alternative substitution of synthetic clinical drugs for the medication of diabetes.

## Introduction

Dipeptidyl peptidase IV (DPP IV), a membrane surface antigen protein, is known as an adenosine deaminase complexing protein 2 or CD26 and involved in immune regulation, signal transduction, and apoptosis related proteins^1^. Recent literature has revealed glucose-dependent insulinotropic peptide (GIP), neuropeptide Y (NPY), glucagon-like peptide (GLP)−1 and 2, and chemokines were all involved in the pathways of glucose metabolism^2^. In pancreatic β cells, the inhibition of DPP IV exerts incretin peptides binding to G protein-coupled receptors (GLP-1R and GIP-R)^3^. DPP IV is ubiquitously found in the capillary endothelium, rapidly inactivates GLP-1 (7–36)-amide in the intestinal capillaries, the hepatoportal vein, and the periphery^4^. DPP IV mediates GLP-1 degradation by cleaving the di-peptide at the N-terminus and removing the histidine-alanine dipeptide, yielding GLP-1 (9–36)-amide^5^. In recent years, DPP IV employs an effective role in the control of blood sugar as oral hypoglycemic agents mostly based on the inhibition of DPP IV^6^. Sitagliptin can raise GLP-1 and GIP (incretin hormone concentrations), respectively, through the inhibition of DPP IV enzyme, as both increase pancreatic cells to synthesize and release insulin. Conversely, the side effects of sitagliptin include nasopharyngitis, upper respiratory tract infection, abdominal pain, and headache^7^. Henceforth, when screening candidates from a natural compounds database from as alternative chemicals with less side effects has become an urgent and interesting issue.

Molecular docking is a fast and accurate prediction method to identify the structures of protein−ligand complexes, which can be used for computations for molecular recognition especially for drug design^8^. It considers all change factors, such as receptors active site, small molecule structure, or ligand binding and then finds low-energy binding modes^9^. In recent years, protein chemistry, nuclear magnetic resonance (NMR), X-ray diffraction, and molecular biology have been developed for molecular docking along with applied software such as AutoDock and DOCK^10,11^. Lately, the approach of computation is advantageous for decelerating the duration of screening at the preclinical stage of drug development. In the present study, experiments were conducted to screen and verify hypoglycemic efficacy for natural compounds based on our chemical database. In this study, the first part (*in silico*) employed a molecular docking (DPP IV, PDB ID: 2ONC as a template) with virtual screening via Discovery Studio to search for natural phenolic compounds. In *in vitro* cell assays and high fat and high fructose diet (with 60% fructose) induced diabetic mice (*in vivo*) were employed to verify the potency of candidate natural phenolic compounds.

## Results

### Molecular docking

**Figure 1:**
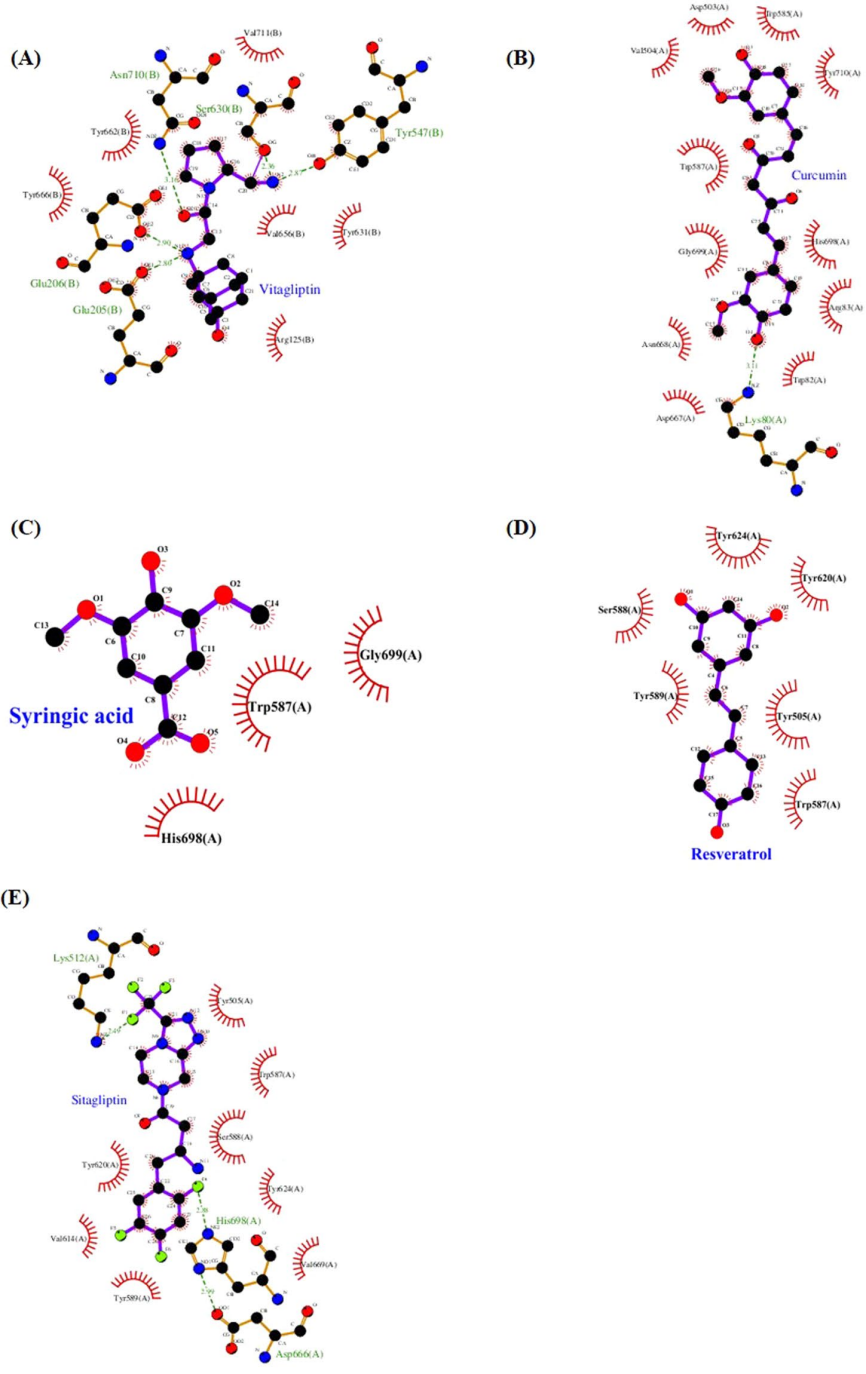
Structure of DPP IV active site and scheme of natural compounds binding to the active site of DPP IV. Simulation of (**A**) DPP IV active site co-complexed with vitagliptin (PDB ID: 3w2t)^40^, (**B**) curcumin, (**C**) syringic acid, (**D**) resveratrol, and (**E**) DPP IV inhibitor (sitagliptin) binding to DPP IV active site. Selected the new DPP IV inhibitor by virtual screening was found from screening compound binding with the active site of DPP IV.

In order to find new DPP IV inhibitors, the structure and active site of DPP IV were searched from the protein data bank (PDB) (Fig. 1A). The active sites that involved in inhibitory efficacy are His740, Ser630, Tyr631, Tyr547, Tyr666, Tyr662, Arg125, Glu205, Glu206, and Phe357. According to chemical structure, the top ten docking candidates could be categorized into three groups as follows: phenolic (curcumin, syringic acid, and resveratrol), flavonoids (catechin, quercetin, and kaempferol), and others (shikimic acid, fumaric acid, tetracosanol, docosanol, camphor, berberine, actinodaphnine, N-methyl-actinodaphne, 16-hydroxycleroda-3,13-dien-15,16-olide (HCD), antroquinonol, and rutin). Based on docking score and PLP2, top ten selected natural compounds were observed as followed: rutin, antroquinonol, HCD, curcumin, quercetin, berberine, syringic acid, and kaempferol. When compared with DPP IV inhibitor-sitagliptin, docking score of top ten selected natural compounds were all higher than that of sitagliptin (Table 1). The inhibitory function of natural compounds based on the intermolecular hydrogen bonding and atomic force was analyzed, curcumin (Fig. 1B), syringic acid (Fig. 1C), resveratrol (Fig. 1D), and DPP IV inhibitor-sitagliptin (Fig. 1E) were selected for further experiments particular in phenolic-curcumin and resveratrol.

**Table 1:** Top fifteen docking candidates of natural compounds with DPP IV (PDB ID: 2ONC).

Compounds	Docking Score	PLP1	PLP2	Numbers of bond
Rutin	79.257	106.45	106.13	3
Antroquinonol	68.479	63.83	62.39	1
16-hydroxycleroda-3,13-dien-15,16-olide	68.29	62.2	59.15	2
Curcumin	66.765	50.87	54.25	3
Quercetin	65.341	49.61	50.88	4
Berberine	65.248	68.54	58.13	1
Resveratrol	63.015	43.93	49.05	2
Syringic acid	62.769	48.98	44.05	1
Kaempferol	61.613	13.41	21.18	3
Catechin	60.648	42.66	48.69	2
Benzyl cinnamate	45.390	62.62	59.18	5
Eugenol	44.988	6.14	30.71	4
Eugenol methyl ester	35.848	45.41	43.28	4
Ethyl gallate	32.145	33.69	33.72	2
2,6-Dimethoxy-1,4-benzoquinone	31.988	38.38	34.88	2
Sitagliptin (DPP IV inhibitor)	57.430	39.59	28.79	3

PLP, pairwise linear potential, represents as a docking energy.

### *In vitro* assay of DPP IV inhibitory effect

#### Effect of selected natural phenolic compounds on DPP IV Enzyme activity

**Figure 2:**
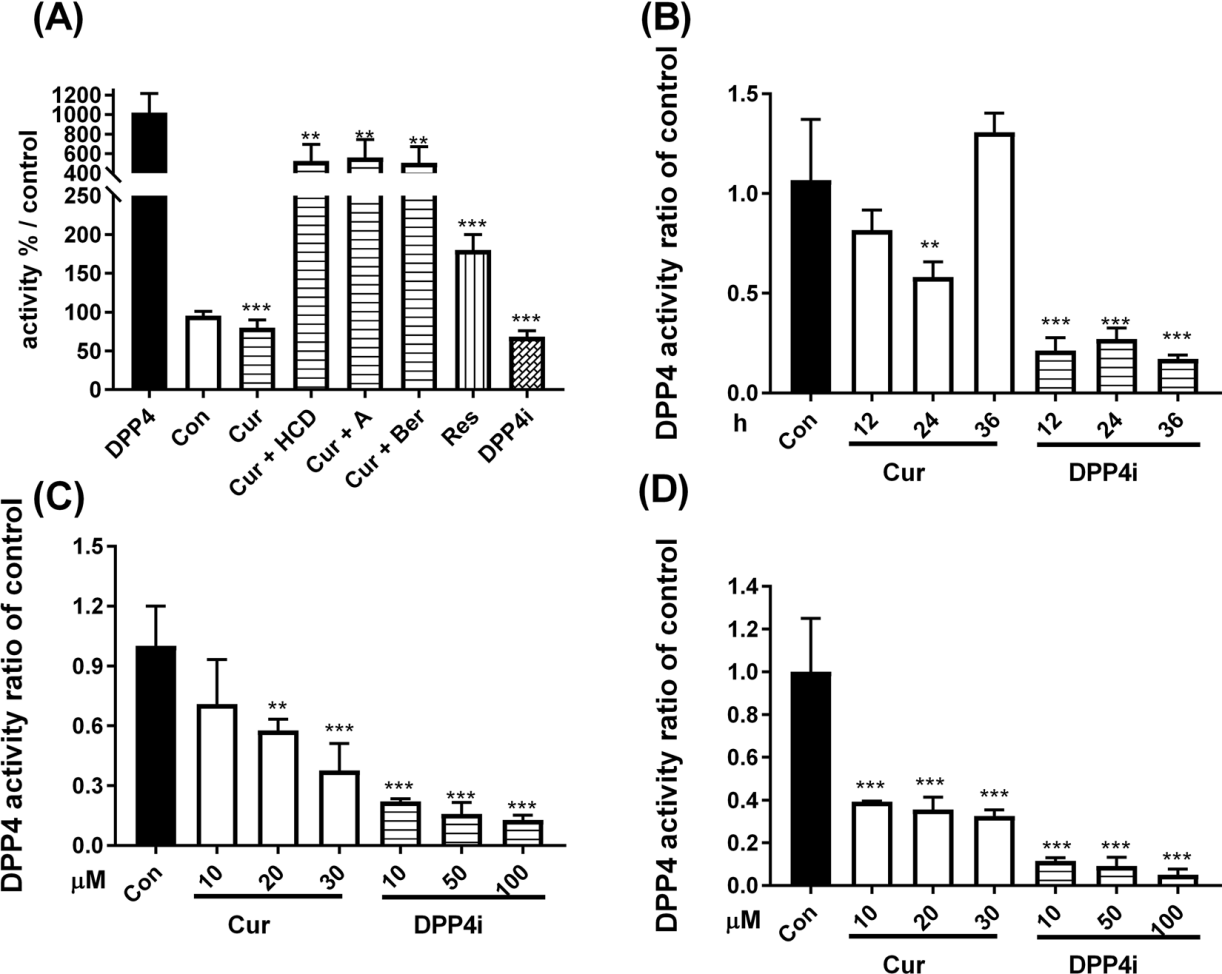
Inhibition of DPP IV activity via natural compounds. The (**A**) enzymatic inhibition of DPP IV activity were determined after treating with P32/98 (Con), curcumin (Cur), resveratrol (Res), sitagliptin (DPP4i), curcumin mixed with berberine (Ber), antroquinonol (**A**), and 16-hydroxycleroda-3,13-dien-15,16-olide (HCD), respectively. (**B**) In Caco-2 cells, time-dependent with 10 μM of curcumin (Cur) and 10 nM of sitagliptin (DPP4i) for 12, 24, and 36 h, respectively. And dose-dependent with 10, 20, 30 μM of Cur and 10, 50, and 100 nM of DPP4i for (**C**) 12 h and (**D**) 24 h manner in Caco-2 cells, respectively. Data are expressed as means with standard deviations (mean ± SD). **P < 0.01; ***P < 0.001 vs. DPP IV (**A**) or control (**B**–**D**) with one-way ANOVA.

To test the DPP IV-inhibitory effect of natural phenolic compounds directly, 100 µM of resveratrol and curcumin, 10 nM of DPP4i and known inhibitors P32/98 were used. The results revealed that curcumin and resveratrol had inhibitory effects. The inhibitory rate of curcumin was up to 50%, which was higher than for P32/98 and resveratrol (Fig. 2A). To validate the results of *in silico* screen, the enzymatic assays of top-4 selected natural compounds were measured. In enzymatic assay, inhibitory efficacy of selected compounds is highly according to the order of docking result except rutin (Table S1). Furthermore, to understand the synergistic effect of DPP IV inhibitory activity in curcumin and other potent candidates, curcumin mixed with HCD, antroquinonol, and berberine were used to test the inhibition rate of DPP IV activity. The results revealed lower inhibition rates for the curcumin mixture than for curcumin alone (Fig. 2A), suggesting the inhibitory effect of these potent DPP IV inhibitor candidates might act in a competitive manner. According to the abovementioned results, curcumin was selected for further experiments.

#### Natural compounds inhibited DPP IV protein production

To examine the DPP IV inhibitory effect of curcumin in time-course and dose-dependent fashions, Caco-2 cells were treated with various concentrations of curcumin and DPP4i for 12, 24, and 36 h, respectively. In a time-course manner, DPP IV activity was significantly decreased after 24 h of 30 μM curcumin treatment (Fig. 2B). Additionally, the inhibiting DPP IV activities of top-4 selected compounds in Caco-2 cells were associated with *in silico* screen and in enzymatic assay, and rutin was the weak response in both *in vitro* enzymatic and cell assays (Table S2). After curcumin treatment for 12 h, 20 and 30 μM of curcumin inhibited DPP IV activity (Fig. 2C). Likewise, all concentrations of curcumin inhibited DPP IV activity in 24 h treatment (Fig. 2D). According to the above results, curcumin could reduce DPP IV activity and inhibitory activity prolonged up to 24 h.

#### Effect of selected natural compounds on the phosphorylation ERK

**Figure 3:**
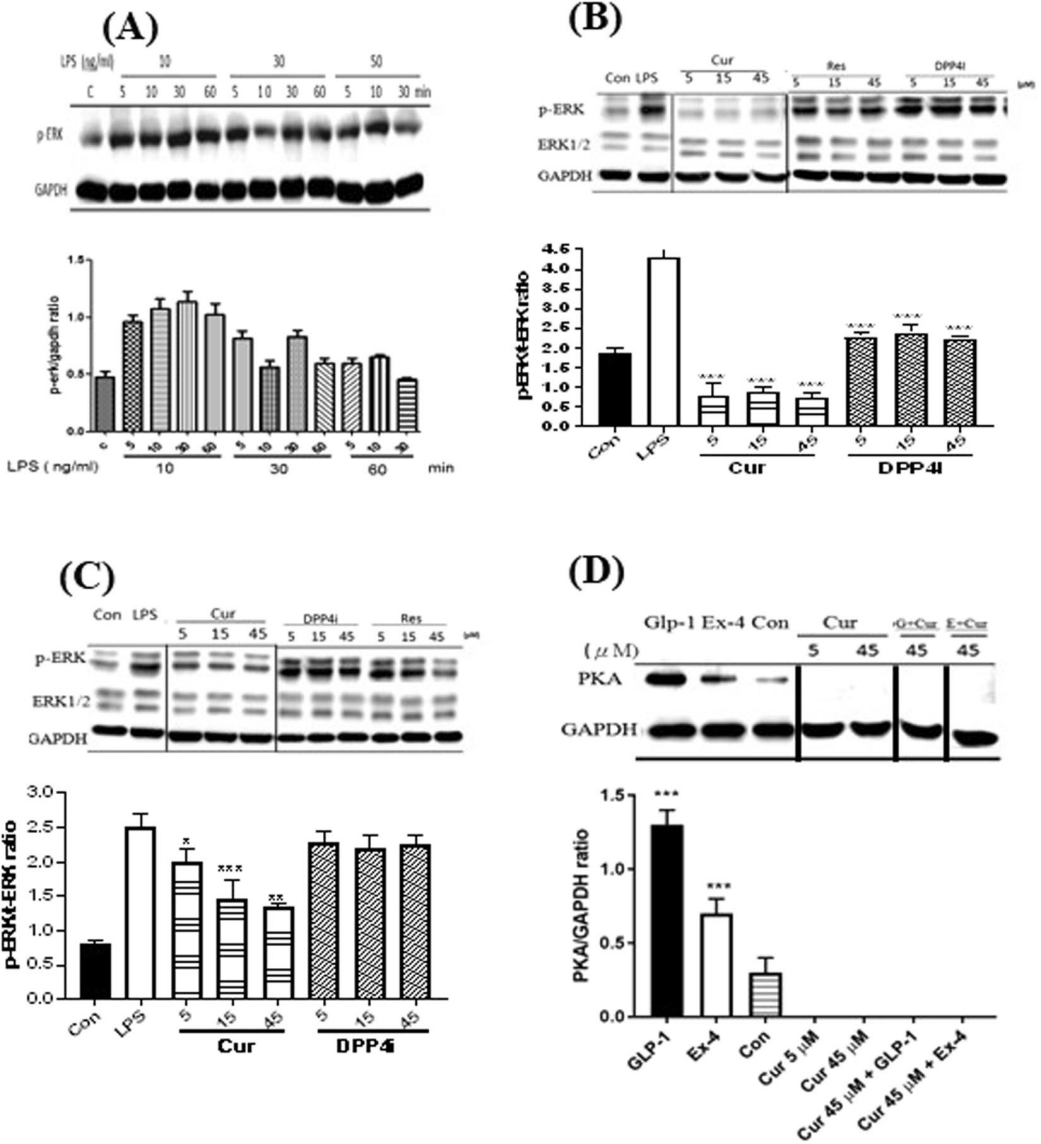
The inhibition of LPS-induced ERK phosphorylation and GLP-1-induced PKA production by curcumin. The protein levels of phosphorylated ERK after (**A**) 10, 30, and 60 min of LPS (10, 30, and 50 ng/mL) stimulation, (**B**) 10 and (**C**) 30 min of 10 ng/mL LPS stimulation followed by various concentrations of 5, 15, and 45 μM curcumin (Cur) and 5, 15, and 45 nM sitagliptin (DPP4i) treatment, respectively. (**D**) AR42J cells were treated with 1 nM glucagon-like peptide-1 (GLP-1), 1 nM exendin-4 (Ex-4), and mixture with 45 μM of curcumin (Cur) for 48 h. Protein expression levels were determined by Western blot. ERK, PKA, and GAPDH were stained and exposed from the same gel which cut into appropriate length after transferring. Data are expressed as means with standard deviations (mean ± SD). *P < 0.05; **P < 0.01; ***P < 0.001 vs. LPS with one-way ANOVA.

To estimate the time of LPS exposure, C2Cl2 cells were treated with 10, 30, and 50 ng/mL LPS for 5, 10, 30, and 60 min, respectively; and analyzed phosphorylated and total ERK protein levels. In 30 and 50 ng/mL of LPS treatment, the phosphorylation ERK failed to change with time, which might cause by cell injury induced by high concentration of LPS. Also, p-ERK/t-ERK did not differ between 30 and 60 min of 10 ng/mL LPS treatment (Fig. 3A). Subsequently, using 10 ng/mL of LPS stimulation for 10 and 30 min conditions as a model was employed to analyze p-ERK/t-ERK ratio after treatment for 5, 15, and 45 µM of curcumin. In 10 min of LPS stimulation, all concentrations of curcumin effectively reduced the ratio between phosphorylated ERK and total ERK (Fig. 3B). Furthermore, curcumin inhibited ERK phosphorylation in 30 min of LPS stimulation but DPP4i could not, which was an additional event and proved higher DPP IV-inhibitory activity for curcumin than for DPP4i (Fig. 3C).

#### Effect of natural compounds on Protein kinase A (PKA)

Therefore, the protein level of PKA in GLP-1 stimulated cells can be a downstream marker of DPP-4 activity and the PKA level was measured after treatment of GLP-1 or Ex-4 combined with curcumin. The protein level of PKA was significantly increased after GLP-1 or Ex-4 treatment, which indicated the PKA level, could be increased by GLP-1 or Ex-4 induction. After curcumin treatment, PKA protein level was decreased whether GLP-1 or Ex-4 co-treatment (Fig. 3D). This result indicated that curcumin could not inhibit DPP IV activity in pancreatic cells.

### *In vivo* test of potent DPP IV inhibitor

#### Hypoglycemic and weight controlling ability of curcumin *in vivo*

**Figure 4:**
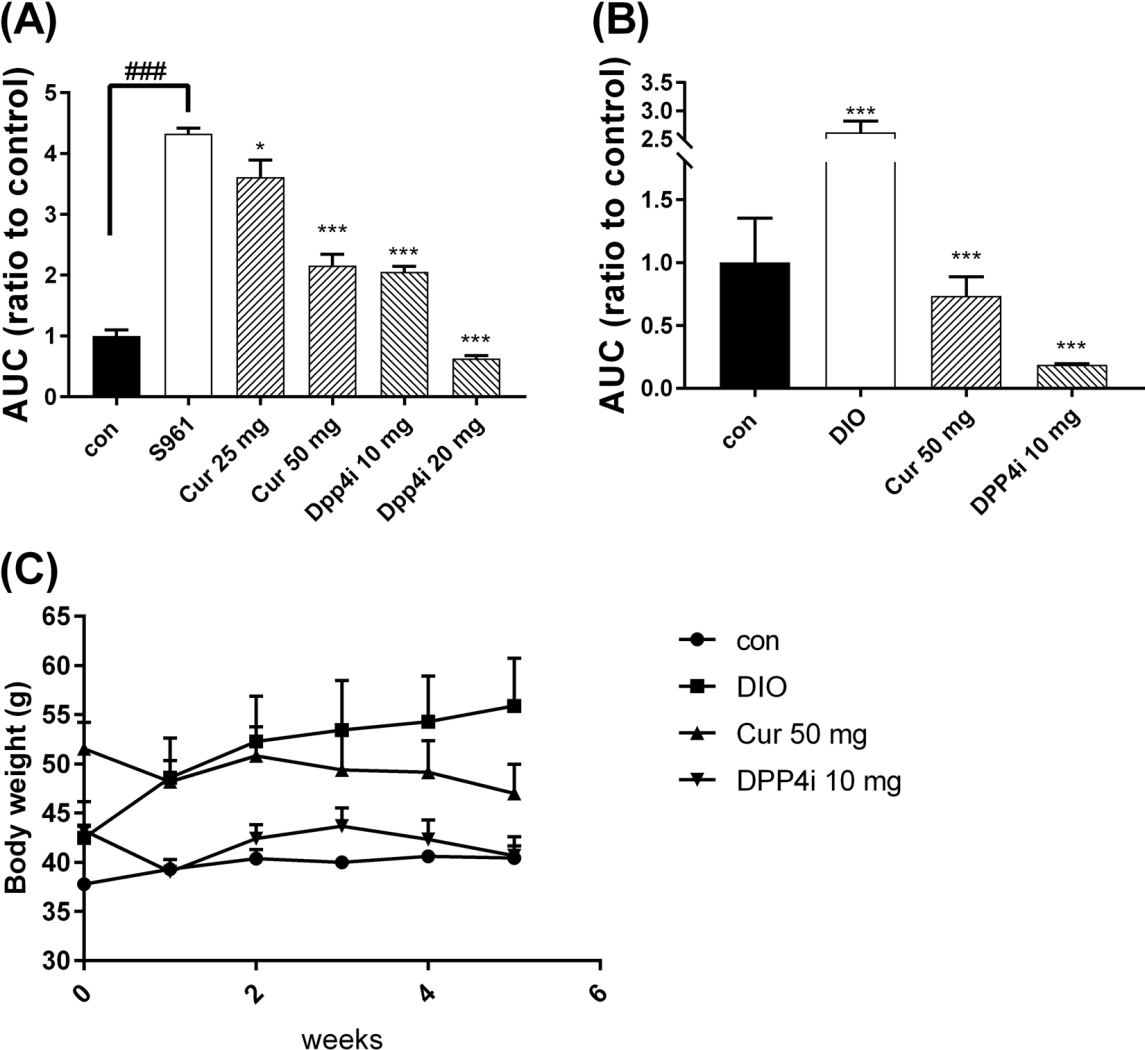
The blood glucose and body weight change in oral administration of curcumin. Blood glucose was measured after (**A**) short-term and (**B**) long-term (5 weeks) treatment of curcumin. (**C**) Body weight was also measured during 5-weeks of curcumin treatment. Con (Control), Cur (Curcumin), DIO (diet-induced obese), and DPP4i (Sitagliptin, DPP IV inhibitor). Data are expressed as means with standard error (mean ± SE, n = 5). ***P < 0.001 treatments vs. the control with one-way ANOVA. ^###^*P* < 0.001 Dio vs. the control with T-test.

To test the hypoglycemic function of curcumin, an oral glucose tolerance test (OGTT) on C57BL/6 mice was conducted and combined with an S961 i.p. injection. After S961 treatment, the AUC of the S961 alone group was significantly higher than for the untreated control. Besides, AUC was decreased by oral gavaging 50 mg/kg B. wt. of curcumin (Fig. 4A). This result elicited the hypoglycemic potential of curcumin.

The induced high blood sugar mice (HBL) were confirmed after GT and IGT test and next were treated with curcumin and DPP4i for 5 weeks, respectively. The body weight of mice was determined every week during treatment and carried out OGTT in the end. From the OGTT, we observed in the diabetic mice for long-term treatment with natural compounds, the data showed that the treatment group had significantly lower AUC levels than the non-treatment group at 180 min (Fig. 4B). Also, the diabetic mice were divided into treatment and non-treatment groups and normal mice were used as a control and were used to test curcumin efficacy for 5 weeks (Fig. 4C). In diabetic mice from the treatment group, average weight loss was 4–6 g; whereas, the body weight of the non-treatment group continued to increase. In this study, a high fat diet and high fructose diet was applied to induce diabetes and according to the experimental results, the diabetic mice had insulin resistance, which caused an excess of free fatty acids. Insulin resistance reduces the sensitivity of insulin to increase triglyceride hoarding and body weight. After treatment, blood sugar and body metabolism were ameliorated and consequently insulin resistance levels decreased further from the lowing of free fatty acids and triglyceride storage, which achieves a decrease in body weight to improve obesity.

#### Analysis of serum biochemical values

**Figure 5:**
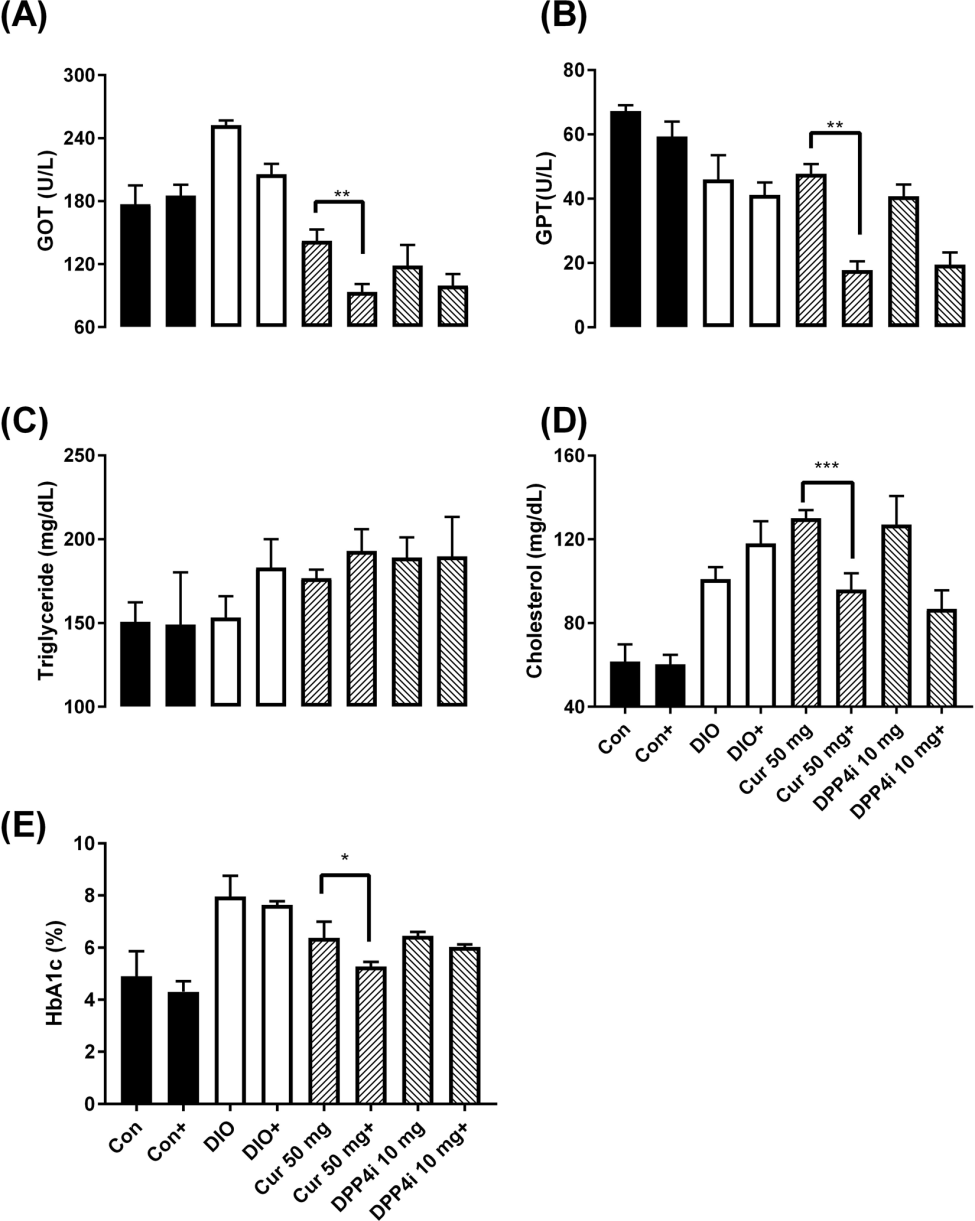
The change of blood biochemical value for long-term treatment with natural compounds in diabetic mice. (**A**) Glutamate oxaloacetate transaminase (GOT), (**B**) glutamate pyruvate transaminase (GPT), (**C**) triglyceride (TG), (**D**) cholesterol (CHO), and (**E**) glycated hemoglobin (HbA1c) levels were measured after five weeks of treatments. Con (normal), DIO (diet-induced obese), 50 mg/kg B. wt. Cur (Curcumin), and 10 mg/kg B. wt. DPP4i (Sitagliptin, clinical DPP IV inhibitor). + : after treatment. Data are expressed as means ± standard error (mean ± SE, n = 5) with pair T-test. *P < 0.05; **P < 0.01 vs. each group prior to treatment.

After five weeks of treatment, blood was collected by piercing the cheek and serum biochemical values (GOT, GPT, TG, CHOL, and HbA1c) were analyzed. Compared before and after the mice were given natural compounds, all blood biochemical values were significantly reduced except CHOL (Fig. 5). The blood biochemical values and body weight changes showed that curcumin could regulate blood sugar and body weight with no adverse effects in the liver.

## Discussions

Type 2 diabetic mellitus (T2DM) has become a global epidemic that is caused by obesity, insulin resistance, and dyslipidemia. For controlling T2DM, stabilization of blood sugar levels is the main task to prevent diabetic symptom deterioration and further cause the diabetic complications. Two distinct gut-derived peptide hormones GLP-1 and gastric inhibitory peptide (GIP) potentiate glucose could stimulate insulin secretion^12^. GLP-1 exhibits anti-diabetic effects include potential central anorexic effects, impairment of glucagon secretion, and inhibition of gastric emptying^13^, but is rapidly degraded by DPP IV, a proline-containing peptide. To identify or develop DPP IV inhibitors for the treatment of T2DM is the current main topic^14^. Molecular docking was employed to find natural compounds that have inhibitory activity on DPP IV. The crystal structure of DPP IV was obtained from the Protein Data Bank (PDB code: 2ONC)^15^. According to the 3-D structure, it was found that DPP IV had three major parts for ligand binding such as S1 exist in catalytic residues, and S2 and S3 with ionic interaction sites. Hereafter, the different bind space may cause a structural change of the design inhibitor. The S1 bind site is more stable for binding while S2 and S3 need stronger bonds to combine the effect^16^. The active sites of DPP IV are His740, Ser630, Tyr631, Tyr547, Tyr666, Tyr662, Arg125, Glu205, Glu206, and Phe357^17^ by literature search. We have selected parameter-PMF (H-bond and atomic force) and binding active sites as the main basis for natural compound screening and presented the binding energy by PLP2 according to our previous study^18^. The top five high docking score compounds including rutin, antroquinonol, quercetin, curcumin, and HCD for further experiments. In the other reports, AutoDock software was used to find a new DPP IV inhibitor^19^, compared to this study, against DPP IV active site in S1 pocket, but they selected parameter–PLS to screen the compounds. The different main bonding caused distinctive characteristics of candidate inhibitors. Clinical drugs such as DPP IV inhibitor are Sitagliptin (Januvia, Merck), Vildagliptin (Glavus, Novartis), and Alogliptin (Nesina, Takeda)^20^. They have similar structures such as amino-like linkages, cyanopyrrolidine moieties, and xanthene/pyrimidine moieties^19^. In this study, natural compounds were identified by virtual screening and they had similar structures. The most convenient tool for drug design and development is virtual screening, but further experiments are needed to confirm efficacy.

There are many Chinese traditional herbal medicines have been used to treat DM with unclear functional components and mechanism. The literature has demonstrated that grape seed-derived procyanidins (GSPE) can inhibit DPP IV activity, down-regulate gene expression in the intestines, and increase the plasma insulin/glucose ratio in response to orally administered glucose^21^. Our molecular docking results showed that screened natural compounds can inhibit DPP IV activity. In literatures, short-term treatment of phenylcyclohexyl acetic acid could decrease blood sugar levels in DIO mice and long-term gavaging also shows lower the AUC of glucose by 51% in IGTT with unknown mechanism^22^. Some natural compounds reduce insulin resistance by the activation of AMPK like Epigallocatechin gallate^23^; the activation of PPAR-gamma such as Luteolin^24^; and unknown mechanism like Daidzein^25^. To date, Chinese and Indian turmeric has been used in multiple functions for long periods and demonstrated well for anti-cancer, anti-oxidation, and inflammation^26^. Curcumin is extracted from root of *Curcuma longa* Linn. Some articles have also indicated that curcumin exhibited lower blood sugar function in animal model and human trials^27^. Previous study represented that (arene) Ru(II)-curcumin complex could inhibit DPP IV activity via molecular docking and *in vitro* enzymatic assay^28^. In the present results further confirmed that naked curcumin could inhibit DPP IV activity in *in silico*, in enzymatic assay, *in vitro cells* and *in vivo* assays. In another previous study, resveratrol has shown DPP IV inhibitory activity in *in vitro* enzymatic assay^29^. Again, our study provides further evidence to confirm the inhibitory ability of resveratrol toward DPP IV not only in *in vitro* enzymatic, but also in *in vitro* cell test and *in vivo* animal test. When compared with docking scores of rutin, HCD, and antroquinonol are higher than that of curcumin. The preliminarily inhibitory potency among them in cell level was evaluated that they are nicely according to the results *in silico* and in enzymatic assay (data not shown).

The animal model of DM can be divided into chemically-induced (CIDM) and diet-induced (DIDM). Glucosamine, dexamethasone, streptozotocin (STZ), STZ with nicotinamide, and alloxan were often applied in chemical inductions^30^, which destroy β cells and rapidly showed symptom of T1DM. However, the disadvantage of chemical induction frequently leads animals to death. In this study, T2DM mice were induced with long-term intake of high-fat and high-fructose diet^31^ because diet-induced approach was similar with the habit of DM patients. The IGTT was employed to check insulin resistance in DIO-mice^32^ and the results confirmed successful T2DM induction. In long-term treatments, the body weight of mice was decreased compared with non-treated group which was continuously induced (Fig. 4C). These results also revealed weight-loss and hypoglycemic effect in DIO mice, which exhibited potential in anti-diabetic drugs development.

Generally, long-term drug treatment may cause hepatotoxicity, hence, hepatotoxicity of long-term treatment of curcumin was investigated. Curcumin had significantly reduced GOT and GPT (indicator of liver function^33^), which demonstrated no chronic hepatotoxicity in curcumin (Figs 5A and 5B). High-fat diet is induced concomitant phospholipid decreased in the liver subsequence TG levels increase^34^. In this study, curcumin could result in lower TG levels but not CHO level, which exhibited hypolipidemic effect of curcumin (Figs 5C and 5D). HbA1c is the indicator of long-term blood sugar levels which is based on the lifetime of red blood cells^35^. HbA1c levels in DIO mice after long-term curcumin treatment showed stabilizing ability of curcumin in blood sugar levels (Fig. 5E). Curcumin could inhibit of NF-κB (activator of MAPK/ Wnt/β-catenin phosphorylation and inhibitor of ERK phosphorylation) and subsequent with HbA1c reduce. In our diet-induced diabetes study confirmed that curcumin could alleviate insulin resistance and promoted the metabolism to control body weight, which is the golden method for diabetic patient therapy^36^.

The potent natural DPP IV inhibitors could be firstly screened via molecular docking. At enzymatic and cellular level, curcumin, the most potent phenolic candidate in enzymatic assay, can inhibit DPP IV activity in Caco-2 cells and LPS-induced ERK phosphorylation in C2C12 cell. *In vivo* glucose tolerance revealed that curcumin administrated blood sugar levels in short-term or long-term scenarios. Moreover, the changes of body weight and blood chemical values after long-term treatment showed weight loss in high fat and high fructose-induced diabetic mice with an absence of liver intoxication. Taken altogether, natural DPP IV inhibitors could be screened via computational biology and curcumin exerts a potent hypoglycemic agent for diabetic treatments.

## Materials and Methods

### Molecular docking

#### Natural compounds

Natural compounds were provided by Dr. Yi-Chen Chia (Department of Food Technology, Tajen University, Pingtung, Taiwan) and total synthesis of antroquinonol was obtained from Dr. Chin-Piao Chen (Department of Chemistry, National Dong-Hwa University, Hualien, Taiwan).

#### Structure modeling of Dipeptidyl peptidase IV

3-D structure of the DPP IV protein (PDB ID: 2ONC) was obtained from the Research Collaboratory of Structural Bioinformatics (RCSB) in Protein Data Bank and modeled by Accelrys Discovery Studio (DS; Accelrys Software Inc., San Diego, CA, USA). The 3-D structures of DPP IV inhibitor were also extracted and transferred into the modeled structure to use it as a guide for docking studies.

#### Pharmacophore generation

Models of all molecules were built and hydrogens are added, then the structures were minimized in the minimization module in Discovery Studio. According to chemical characteristics such as hydrophobic, hydrogen bond donor, hydrogen bond acceptor, and ring aromatic; and then choose a feature to calculate the scores for binding strength were described in detail as our previous study^18^ with minor modification. In this study, the active sites in DPP IV enzyme were located at His740, Ser630, Tyr631, Tyr547, Tyr666, Tyr662, Arg125, Glu205, Glu206, and Phe357^15^ and calculated the scores of analog chemical molecules in conjunction with these binding sites using Discovery Studio and compared various bonding methods to determine the feasibility of candidate inhibitors.

### *In vitro* DPP IV inhibitor screening

#### Cell culture

Mouse muscle myoblast cell line C2C12, rat pancreatic tumor cell line AR42J, and human colorectal cancer cell line Caco-2 were all obtained from Bioresource Collections and Research Center (BCRC, Hsinchu, Taiwan). Three cells were cultured in high-glucose DMEM (Thermo-Fisher, Waltham, MA, USA) supplemented with 10% (C2C12) or 20% (AR42J and Caco-2) FBS (Thermo-Fisher) and 1% penicillin/streptomycin (PS, Thermo-Fisher) in CO_2_ incubator (Thermo-Fisher) with 37 °C and 5% CO_2_. The culture media were changed every 2 d. Cells were detached by 0.25% trypsin/EDTA (Thermo-Fisher) for experiments as the cells reached 80% confluence. All experiments were obtained within 20 passages concerning uniformity and reproducibility.

#### DPP IV enzyme activity assay

The DPP IV enzyme activity assay was carried out by DPP IV/CD26 Assay Kit for Biological Samples (Enzo Life Sciences, Farmingdale, NY, USA) and the protocol was followed the menu of company provided. In brief, 50 μL of assay buffer were mixed with DPP IV and tested compounds (curcumin, resveratrol), two mixtures, and DPP4i in 96-well ELISA plate, respectively. Next, H-Gly-Pro-pNa solution was added into the wells and the optical intensity at 405 nm was determined by Opsys MR ELISA reader (Thermo-Fisher).

#### Western blotting

The process of Western blotting was followed the protocol in literature^37^. The blots were pulsed with Western Lightning^TM^ Plus-ECL (Perkin-Elmer Life Sciences) and the signals were determined by the intensity of chemiluminescence by LAS-3000 imager (Fujifilm, Tokyo, Japan).

#### *In vitro* DPP IV Inhibition Assay

Caco-2 cells were seeded into 12-well plate and cultured in 37 °C, 5% CO_2_ overnight for confluence. Next, curcumin (10, 20, and 30 μM) and sitagliptin (10, 50, and 100 nM) were added into wells and incubated for 12, 24, and 36 h, respectively. The treated cells were lysed and the protein level of DPP IV were detected by Western blotting as described prior to.

#### ERK phosphorylation analysis in lipopolysaccharide (LPS)-stimulated muscle cells

The ERK phosphorylation induced by LPS stimulation was described as previous study^38^. Briefly, 5 × 10^4^ per well of C2C12 cells were seeded in a 24 well plate and incubated in culture condition for 12 h to determine the concentration and time course of LPS. Next, cells were stimulated with 10 ng/mL LPS for 10 and 30 min, followed by incubation with 5, 15, and 45 μM of Curcumin for an additional 12 h. The ERK phosphorylation was determined by Western blotting described in a previous section.

#### PKA activation assay in incretin-induced pancreatic cells

PKA activation by GLP-1 was previously described^39^. In brief, 1 × 10^5^ per well of AR42J cells were seeded in a 12 well plate and incubated in culture of condition for 12 h. Next, cells were treated with 1 nM of GLP-1, 1 nM of exendin-4 (Ex-4), 5 and 45 µM of curcumin, 1 nM of GLP-1 with 45 µM of curcumin, and 1 nM of Ex-4 with 45 µM of curcumin for 48 h, respectively.

### *In vivo* assay of potent DPP IV inhibitor

#### Animals

Animal experiments were approved by the National Dong-Hwa University Animal Ethics Committee and were used according to the “Guide for the Care and Use of Laboratory Animals” of National Dong-Hwa University. Male C57BL/6 (8 weeks’ old) and ICR (6 weeks’ old) mice were obtained National Laboratory Animal Center (Taipei, Taiwan) and kept at controlled environmental conditions with room temperature (22 ± 2 °C) and humidity (50 ± 10%). The 12 h light/dark cycle (0600 am–1800 pm) was maintained throughout the study. Mice were fed as commercial diet and water ad libitum.

#### Glucose intolerance induce

ICR mice were allotted into 2 groups. The diet-induced obese group (DIO, n = 15) was fed a high-fat diet (150 g of lard added into 1 kg of commercial diet) and 60% fructose solution for 14 weeks; and the control group (Con, n = 5) was fed with normal diet and water.

#### Glucose tolerance test (GT) and insulin-glucose tolerance test (IGTT)

Mice were fasting for 12 h prior to blood sampling by venipuncture from tail vein. 2 g/kg B.wt. of glucose solution were oral gavage (p.o.) to mice followed by blood sampling every 30 min until reaching 180 min. Blood sugar were analyzed by Accu-Chek glucose analyzer (Roche, Basel, Basel-Stadt, Switzerland) and high blood sugar was defined as which was higher than 200 mg/dL at 120 min after p.o. glucose.

The protocol of IGTT was slight modification of GT. Briefly; 0.8 IU/kg of insulin was p.o. to mice.

#### OGTT

The method of OGTT was similar with GT with small modification. In brief, mice were intraperitoneal injected 40 nmol/kg B. wt. of S961 (insulin receptor antagonist, Novo Nordisk A/S, Kalundborg, Denmark) prior to glucose solution mixed with curcumin (25, 50 mg/kg B.wt.) or DPP4i (10, 20 mg/kg B.wt.).

#### Long-term administrations

Control and DIO ICR mice were subjected to 4 treatments, Con (control), DIO (diabetes-induced obese), Cur (curcumin 50 mg/kg B.wt.), and DPP4i (10 mg/kg B.wt.) for 5 weeks. Body weight of each group was measured every week, and OGTT was carried out after treatment.

#### Blood biochemical value analysis

Blood biochemical value analysis was carried out by submandibular blood collection before and after 5-weeks’ treatment. 300 µL of whole blood sample was separated into 2 portions: 100 µL for glycated hemoglobin (HbA1c) analysis, and the remain was centrifugal at 888 × g, 4 °C for 15 min and serum was used to analyze triglyceride (TG), total cholesterol (CHO), glutamate oxaloacetate transaminase (GOT) and glutamate pyruvate transaminase (GPT) by automatic analyzer (ARTAX Menarini Diagnostics, Florence, Italy) with enzymatic colorimetric assay reagent strips (Human, Wiesbaden, Germany).

### Statistical analysis

All data were expressed as means with standard deviations (mean ± SD) and the data were analyzed using one-way ANOVA with Tukey’s test. Statistical significance was defined as p < 0.05. All statistical procedures were performed with GraphPad Prism version 5.01 (GraphPad Software, Inc., La Jolla, CA, USA).

## Supplementary information

Supplementary tables ans figures
