# Supplementary material for: Natural phenolic compounds potentiate hypoglycemia via inhibition of Dipeptidyl peptidase IV
Source: Sci Rep. 2019 Oct 30;9:15585. doi: 10.1038/s41598-019-52088-7 (PMC6821704; doi:10.1038/s41598-019-52088-7)
Supplement: Supplementary file 1 — Supplementary tables ans figures [file 41598_2019_52088_MOESM1_ESM.pdf]

Research Article

Natural phenolic compounds potentiate hypoglycemia via inhibition of Dipeptidyl peptidase IV

Po-Kai Huang[1], Shian-Ren Lin[1], Chia-Hsiang Chang[1], May-Jwan Tsai[2], Der-Nan Lee[3], Ching-Feng Weng[1,4]

[1] Department of Life Science and Institute of Biotechnology, National Dong Hwa University, Hualien, 97401, Taiwan

[2] Neural regeneration Laboratory, Neurological Institute, Taipei Veterans General Hospital, Taipei, 11217, Taiwan.

[3] Department of Biotechnology and Animal Science, National Ilan University, Ilan, 26047, Taiwan

[4] Faculty of Applied Sciences, Ton Duc Thang University, Ho Chi Minh City, Vietnam

Correspondence: Ching-Feng Weng

E-mail: cfweng@gms.ndhu.edu.tw

TEL: +886-3-8633637

FAX: +886-3-8630255

Supplementary information

The contiguous membrane image could not be provided due to our Western blotting process. After transferring, membrane would be cut immediately based on the molecular weight, hybridized with antibodies, and snapshotted each by each. Therefore, The uncut membrane image was absence.

Figure 3A

I. P-ERK

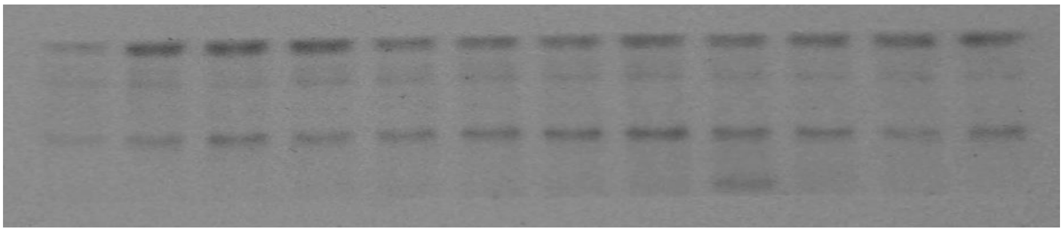

II. **P-ERK**

10 ng/mL 30 ng/mL 50 ng/mL

C 5 10 30 60 5 10 30 60 5 10 30 (min)

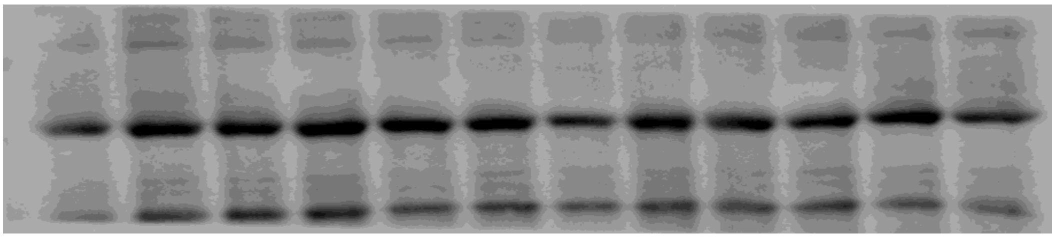

III. P-ERK (This image is used in main figure 3A)

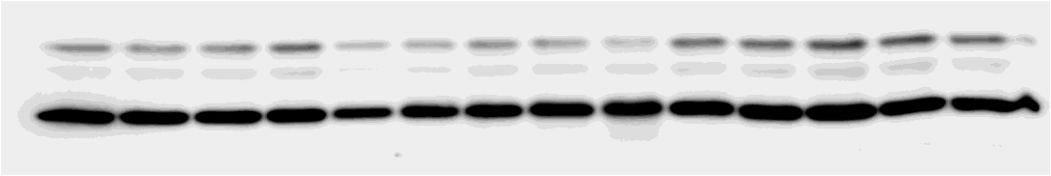

Figure 3B (The rectangular circled zone are used in main figure 3B)

p-ERK

Cur Cur Cur Res

C LPS 5 15 45 5 15 45 5 15 45 5 15 45 (μM)

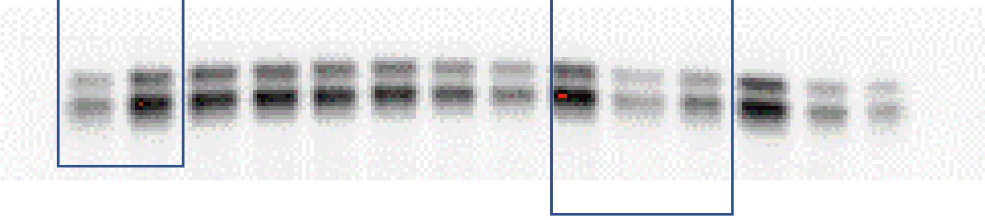

Res Res DPP4i

5 15 45 5 15 45 5 15 45

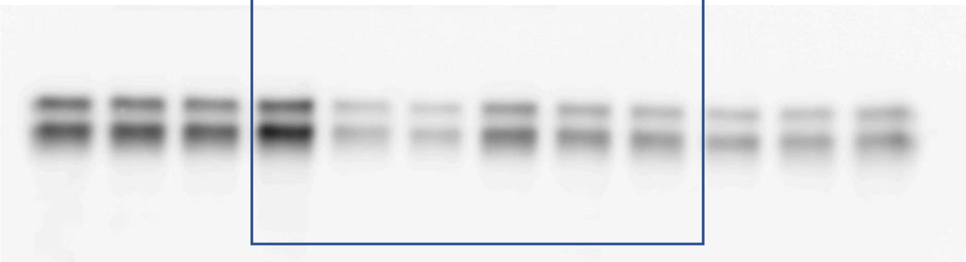

Figure 3C **(The rectangular circled zone are used in main figure 3B)**

P-ERK

Cur Cur Cur

C LPS 5 15 45 5 15 45 5 15 45

(µM)

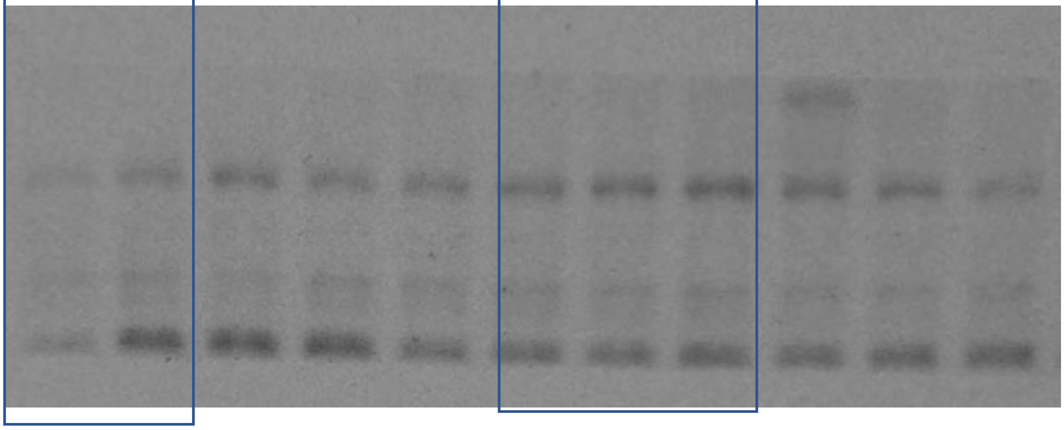

Res Res

5 15 45 5 15 45 (µM)

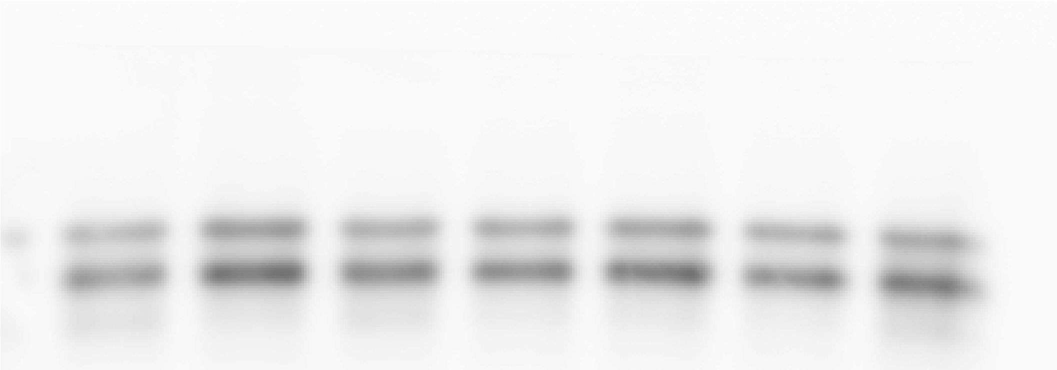

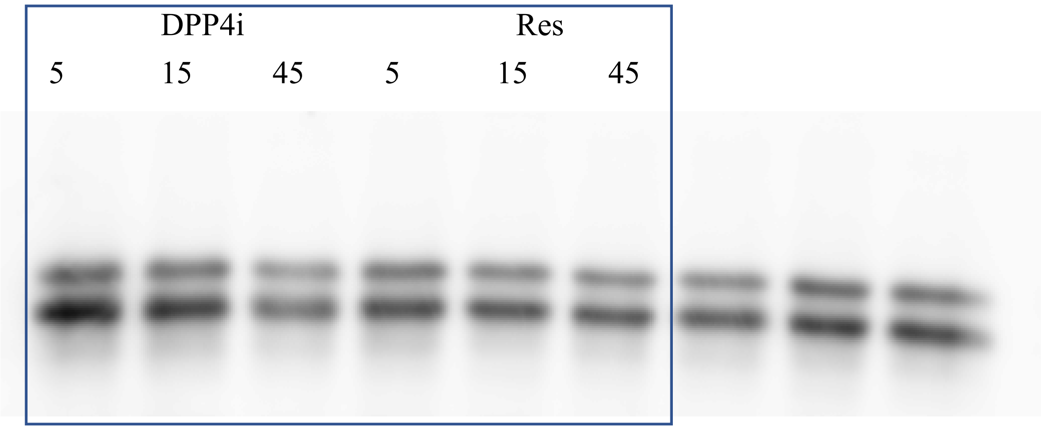

3.

Figure 3D **(The rectangular circled zone are used in main figure 3B)**

I

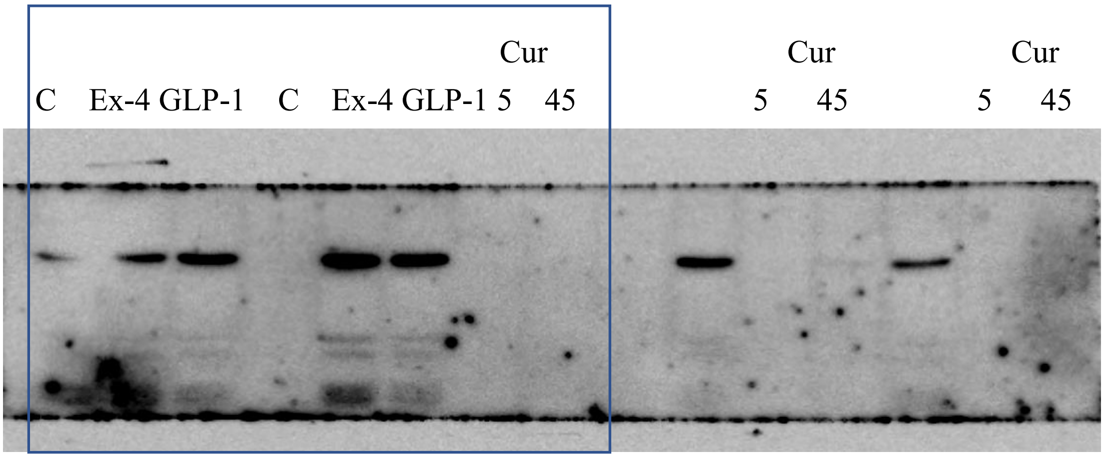

II.

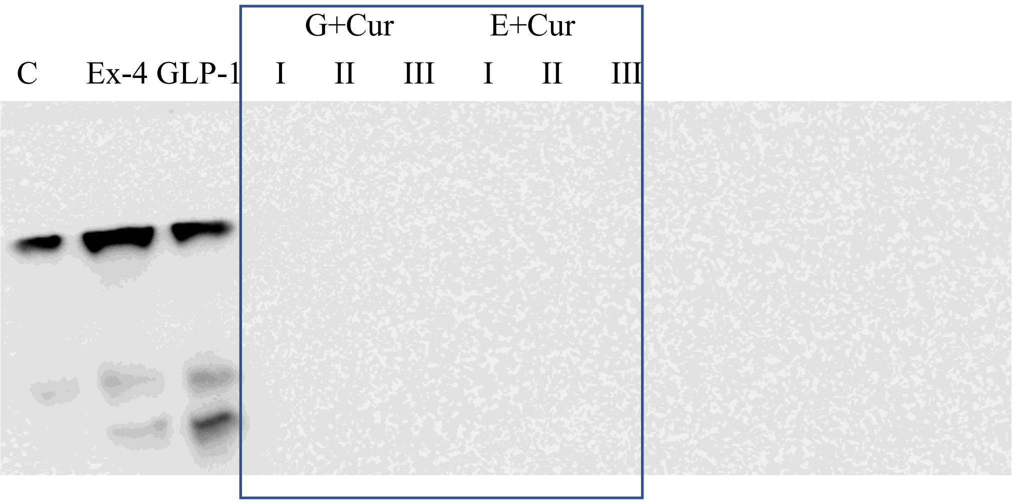

Table S1. Inhibition of DPP-IV activity in top-4 selected natural compounds

Compounds	Concentration (μM)	Relative activity (% to DPP4-IV activity)
**Sitagliptin (DPP-IV inhibitor)**	100	18.3 ±2.4
**Rutin**	100	66.7 ± 6.0
**Antroquinonol**	100	14.6 ± 5.2
**HCD**	100	19.8 ± 3.7
**Curcumin**	100	22.5 ± 4.1

DPP4 activity is 100.0 ± 3.7

Table S2. Inhibition of top-4 selected natural compounds on DPP-IV activity of CaCO-2 cells

Compounds	Concentration (μM)	Relative activity (% to Control)
**Con**	0	100
**Rutin**	30.0	87.2 ± 5.3
**Antroquinonol**	11.5	46.5 ± 6.1
**HCD**	18.8	52.0 ± 4.9
**Curcumin**	30.0	67.1 ± 7.2

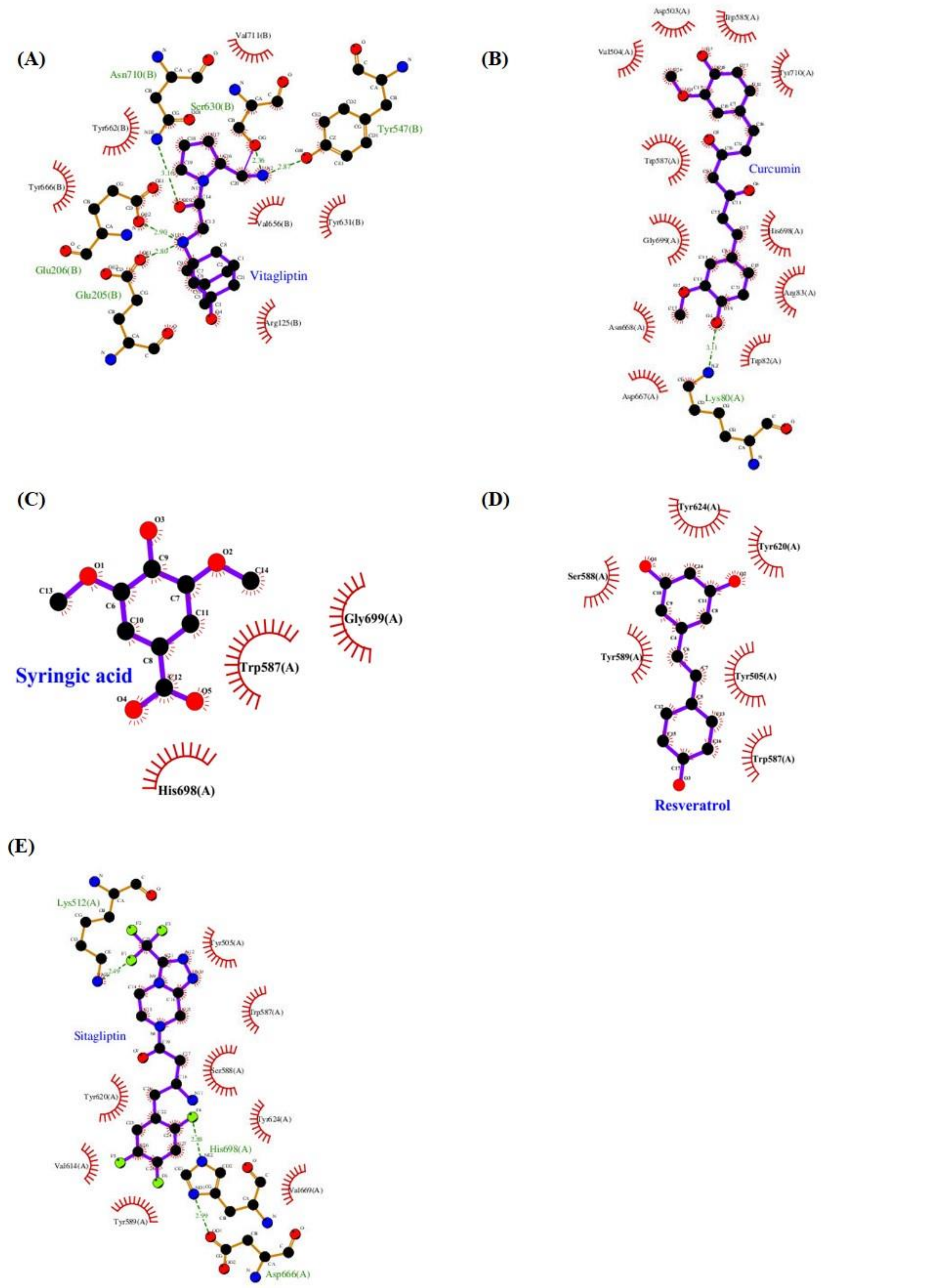

Figure 1. Structure of DPP IV active site and scheme of natural compounds binding to the active site of DPP IV. Simulation of (A) DPP IV active site co-complexed with vitagliptin (PDB ID: 3w2t)[40], (B) curcumin, (C) syringic acid, (D) resveratrol, and (E) DPP IV inhibitor (sitagliptin) binding to DPP IV active site. Selected the new DPP IV inhibitor by virtual screening was found from screening compound binding with the active site of DPP IV.
